# Acute Post-Prandial Cognitive Effects of Brown Seaweed Extract in Humans

**DOI:** 10.3390/nu10010085

**Published:** 2018-01-13

**Authors:** Crystal F. Haskell-Ramsay, Philippa A. Jackson, Fiona L. Dodd, Joanne S. Forster, Jocelyn Bérubé, Carey Levinton, David O. Kennedy

**Affiliations:** 1 Brain, Performance and Nutrition Research Centre, Northumbria University, Newcastle Upon-Tyne NE1 8ST, UK; philippa.jackson@northumbria.ac.uk (P.A.J.); f.dodd@northumbria.ac.uk (F.L.D.); jo.forster@northumbria.ac.uk (J.S.F.); david.kennedy@northumbria.ac.uk (D.O.K.); 2 innoVactiv Inc., Rimouski, QC G5L 9H3, Canada; jberube@innovactiv.com; 3 Institute of Health Policy, Management and Evaluation, University of Toronto, Toronto, ON M4G 4J6, Canada; carey.levinton@utoronto.ca

**Keywords:** seaweed, cognition, cognitive, mood, phlorotannin, phenolic, polyphenol, phytochemical

## Abstract

(Poly)phenols and, specifically, phlorotannins present in brown seaweeds have previously been shown to inhibit α-amylase and α-glucosidase, key enzymes involved in the breakdown and intestinal absorption of carbohydrates. Related to this are observations of modulation of post-prandial glycemic response in mice and increased insulin sensitivity in humans when supplemented with seaweed extract. However, no studies to date have explored the effect of seaweed extract on cognition. The current randomized, placebo-controlled, double-blind, parallel groups study examined the impact of a brown seaweed extract on cognitive function post-prandially in 60 healthy adults (*N* = 30 per group). Computerized measures of episodic memory, attention and subjective state were completed at baseline and 5 times at 40 min intervals over a 3 h period following lunch, with either seaweed or placebo consumed 30 min prior to lunch. Analysis was conducted with linear mixed models controlling for baseline. Seaweed led to significant improvements to accuracy on digit vigilance (*p* = 0.035) and choice reaction time (*p* = 0.043) tasks. These findings provide the first evidence for modulation of cognition with seaweed extract. In order to explore the mechanism underlying these effects, future research should examine effects on cognition in parallel with blood glucose and insulin responses.

## 1. Introduction

The relationship between plasma glucose and cognitive function follows an inverted-U dose response curve [1] and as such, it is beneficial to control the amount of glucose available from food in order to avoid large deviations from the optimal required for cognitive functioning. The rate at which glucose becomes available from food is measured by the glycemic index (GI), whereby a high GI results in a rapid increase in plasma glucose, whereas a slower more sustained increase is observed following a low GI food [2]. A number of studies have demonstrated that consumption of a low GI food has beneficial effects on cognition when compared to high GI food (see [3] for systematic review). However, findings in this area are mixed and it is difficult to draw firm conclusions due to differences between the interventions, other than their GI. For instance, the comparison of meals that provide different levels of macro and micronutrients [4], or the inclusion of fruit in one intervention but not the other [5]; all of which have the potential to impact cognition. In addition, although adopting a low GI diet provides a means to control plasma glucose it has the drawback of limiting the types and quantity of food that can be consumed. 

An alternative approach to consuming low GI food is to slow the absorption of sugar from food by inhibiting the digestion of carbohydrates. This is achieved in the treatment of type 2 diabetes through administration of the oral antidiabetic drug acarbose, which inhibits α-amylase and α-glucosidase, the key enzymes involved in the breakdown and intestinal absorption of starch [6,7]. While effective, there are a number of side effects of acarbose including abdominal distention and gas accumulation [8], which make this a less than ideal solution. A potential natural alternative to acarbose is seaweed. *Ascophyllum nodosum* (kelp) is a brown algae belonging to the *Fucaceae* family, which was shown in vitro to inhibit α-glucosidase to a greater extent than acarbose, while inhibiting α-amylase to a lesser extent [9]. Inhibition of α-glucosidase by *A. nodosum* has been replicated using algae from a variety of locations [10,11,12], and similar α-amylase inhibition has also been observed when using a comparable extraction technique [10]. Inhibition of both enzymes has been shown to correlate with total phenolic content [9], and this is supported by the greater inhibition shown by another member of the *Fucaceae* family, *Fucus vesiculosus* (bladder wrack), which has a higher phenolic content than *A. nodosum* [10]. Previous in vitro findings from (poly)phenol-rich berry extracts also support the role of (poly)phenols in this relationship [13,14,15,16]. Of particular interest are phlorotannins, which are almost unique to brown algae. Comparison of phenolic-enriched and phlorotannin-rich extracts of *A. nodosum* revealed equivalent inhibition of α-glucosidase across the two fractions, which was greater than that shown with acarbose. In terms of α-amylase inhibition, this was shown to be substantial following the phlorotannin-rich fraction but lower than that observed following the phenolic-enriched fraction, suggesting phlorotannins may be a suitable intervention for post-prandial glycemic control [12].

Exploration of the effects of a hot water extract containing phlorotannins derived from *A. nodosum* and *F. vesiculosus* (InSea2^®^—innoVactiv, Rimouski, QC, Canada) in vivo revealed a significant reduction in blood glucose and non-significant modulation of insulin response in rats following starch gavage. This extract was also shown to decrease α-glucosidase and α-amylase in a dose-dependent manner in vitro [17]. Recent data also indicates that in mice fed a normal diet, insulin is significantly decreased at 30 min following supplementation with *A. nodosum* and *F. vesiculosus* extracts, as compared to control. A significant reduction in the initial peak in blood glucose was also observed followed by significantly higher levels at 180 min resulting in no effect on overall area under the curve (AUC) [18]. Support for these findings comes from a randomized, controlled trial demonstrating that 500 mg of the same extract, taken 30 min prior to a 50 g carbohydrate load in the form of bread, lowered the insulin incremental area under the curve and increased insulin sensitivity [19]. Importantly, no evidence of increased side effects was observed when compared to placebo over a 3-h time-period after ingestion [19].

Given that stores of glucose in the brain are limited, a steady supply is needed in order to maintain optimal cognitive function. It is thus likely that effects of glycemic regulation will be most apparent following engagement in high cognitive demand, which results in increased depletion of brain stores. The aim of the current randomized, placebo-controlled, double-blind, parallel groups study was to explore the impact of a brown seaweed extract containing phlorotannins (InSea2^®^) on cognitive function when assessed post-prandially following a high-carbohydrate lunch. It was hypothesized that the algae extract would improve cognition when compared to placebo at a number of time-points post-consumption as part of an intense repeated-block paradigm. 

## 2. Materials and Methods

### 2.1. Design

A randomized, placebo-controlled, double-blind, parallel groups design was utilized. All participants attended the laboratory once and received either seaweed extract or placebo. The study was approved by Northumbria University’s Department of Psychology Ethics Committee and was conducted according to the Declaration of Helsinki (1964). The study was registered on clinicaltrials.gov (identifier: NCT03328923).

### 2.2. Initial Screening

Prior to participation, volunteers were required to provide informed consent. Non-smokers who self-reported that they were in good health, with no pre-existing medical condition or illness, for whom English was their first language and with a BMI > 18.5 and <30 km/m^2^ were recruited to take part. Given the potential impact on cognition or the possibility of interaction with the study intervention, volunteers confirmed that they were not habitually taking any dietary/herbal supplements or medication (excluding the contraceptive pill). Participants did not consume caffeine excessively (determined as >500 mg per day, assessed by caffeine consumption questionnaire) and did not have a history of/current diagnosis of drug or alcohol abuse (self-report). They confirmed that they did not have a history (or current incidence) of head trauma, learning difficulties, attention deficit hyperactivity disorder (ADHD), or dyslexia; they did not suffer from frequent migraines (more than once per month) or have a visual impairment that could not be corrected with glasses/contacts. They also confirmed that they were not pregnant, seeking to become pregnant or lactating. Volunteers with a history of intestinal tract surgery, or any food intolerances/sensitivities, including seafood/fish allergies were also excluded from participation.

### 2.3. Participants

Sixty healthy adults (27 males) between the ages of 18 and 65, who reported post-meal drowsiness were recruited through opportunity sampling within Newcastle upon Tyne and the surrounding areas. The inclusion of those who reported post-meal drowsiness was intended to allow a more sensitive backdrop for effects to be observed following lunch consumption. Participants were reimbursed £40 for their participation. All participants gave their written informed consent prior to their inclusion in the study.

### 2.4. Treatment

Participants attended one study visit and received either 500 mg brown seaweed extract (InSea2^®^) or placebo. InSea2^®^ is characterized by a polyphenol content of >20% (chlorogenic acid equivalent) and is subjected to extensive validation before batch release including microbiological contaminants, chemical contaminants and bioassay (minimum inhibitory activity on α-amylase). Full details of treatment composition can be found in Table 1. Treatment and placebo were identical in appearance and were administered in the form of two tablets contained within a sealed foil sachet in order to mask any taste differences and to ensure that participants remained blind to the treatment they had received. Treatments were prepared by innoVactiv and were then randomized by an independent third-party who had no further involvement in the study, using computer generated random numbers. Each participant received the next sequential randomization number.

### 2.5. Cognitive and Mood Measures

All cognitive and mood measures were administered using the Computerized Mental Performance Assessment System (COMPASS, Northumbria University, Newcastle upon Tyne, UK) a software platform for the presentation of classic and bespoke computerized cognitive tasks. This platform has previously been shown to be sensitive to a range of nutritional interventions [20,21,22]. The computerized tests were conducted via a laptop computer with all stimuli randomized across participant and assessment. Progress through the tasks was controlled by the participant, with brief instructions given on-screen before the start of each task. Tasks were presented in the same order on each occasion and, with the exception of the paper and pencil tasks (word recall), responses were made using a response pad. 

#### 2.5.1. Word Presentation

Fifteen words were presented sequentially on screen for the participant to remember at the rate of one every second, with stimulus duration of one second.

#### 2.5.2. Immediate Word Recall 

The participant was allowed 60 s to write down as many of the words as possible. Outcomes were number correct, errors, and number of intrusions from previous lists.

#### 2.5.3. Simple Reaction Time (SRT) 

An upwards-pointing arrow was displayed on the screen at irregular intervals. Participants responded by pressing the response button as quickly as they could as soon as they saw the arrow appear. The task ran for ~3 min (70 stimuli) and the inter-stimulus interval varied randomly between 1 and 3.5 s. Speed of response (ms) was recorded.

#### 2.5.4. Digit Vigilance

A single randomly selected target digit was continuously displayed to the right of the screen. A series of single digits were then presented to the left of the screen at the rate of 150 per minute. The participant was required to press the response button as quickly as possible every time the digit in the series matched the target digit. The task lasted 3 min. Task outcomes were accuracy (%), reaction time for number of correct responses (ms) and false alarms (number).

#### 2.5.5. Choice Reaction Time (CRT)

An arrow appeared on the screen pointing to the left or to the right. Participants responded by pressing a left or right response pad button corresponding to the direction of the arrow. The task ran for ~3 min (70 stimuli) and the inter-stimulus interval varied randomly between 1 and 3.5 s. The outcomes were speed of response (ms) and accuracy (% correct).

#### 2.5.6. Visual Analogue Scales (VAS)

Participants rated their current subjective state by positioning an ‘X’ with a mouse/cursor on lines headed ‘concentration’, ‘mental stamina’, ‘physical stamina’ (anchored at the left by ‘very low’ and the right by ‘very high’) and ‘mental tiredness’ and ‘physical tiredness’ (anchored at the left by ‘not at all’ and the right by ‘extremely’). They also rated their current levels of hunger, thirst and fullness (‘not at all’ and ‘extremely’). Item scores were calculated as % distance along the line from the left.

### 2.6. Procedure

Participants were required to attend the Brain, Performance and Nutrition Research Centre laboratory of Northumbria University on one occasion having abstained from all food and drink, except water, for 12 h. The visit commenced at 10 a.m. and participants left at approximately 4 p.m. They remained in the research center throughout. Testing took place in a suite of testing facilities with participants visually isolated from each other. 

The initial phase of the laboratory visit comprised: obtaining of informed consent; confirmation of eligibility to take part, including completion of a caffeine consumption questionnaire and collection of demographic data; training on the cognitive and mood measures with one full completion of the tasks; randomization to treatment. 

**Figure 1:**
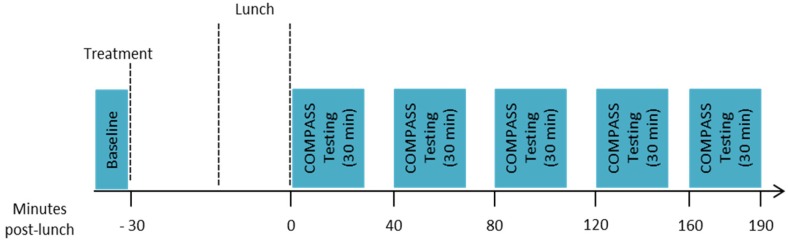
Structure of the testing session.

Participants then performed the baseline assessment (10 min), which comprised one completion of immediate word recall, simple reaction time (SRT), digit vigilance, choice reaction time (CRT) and visual analogue mood scales (VAS). Participants then took their allotted treatment and had a 30-min break before commencing their lunch. Lunch consisted of 4 sweet waffles with 2 tablespoons of pure maple syrup (containing 50 g of carbohydrates as starch and sugar) and 250 mL of water to drink (ad libitum water consumption was also permitted). Participants were given 20 min to eat and were encouraged to eat all of the food provided if possible. All participants conformed to this request. Immediately following lunch their first post-dose assessment commenced, this was identical to the baseline but involved 3 repetitions of the attention tasks (SRT, digit vigilance and CRT) in succession preceded by one repetition of immediate word recall and followed by one completion of the VAS (~30 min). Further identical assessments commenced at 40, 80, 120 and 160 min post-lunch, with ~10-min breaks between each assessment. Assessments were conducted between 0 and 190 min post-meal on the basis of previous data showing reductions in insulin levels across a 180 min post-prandial period following the same seaweed extract [19]. The timings of assessments within that period were dictated by the length of the assessment and the allowance of a short rest between assessments. The structure of the testing session is shown in Figure 1.

### 2.7. Statistical Analysis

Blinded data screening and analysis were conducted on all measures with codes broken only once all analyses were complete. To ascertain comparability between treatment groups, baseline performance and demographics were analyzed; baseline data and continuous demographic variables were analyzed by independent *t*-tests, and categorical characteristics by Pearson’s chi-square. Any demographic characteristics that differed significantly between groups were entered in the analyses of efficacy. 

All attention measures were analyzed with linear mixed models including the respective baseline as a covariate and the terms treatment, assessment, repetition, respective baseline, treatment × assessment, treatment × repetition, treatment × assessment × repetition as fixed effects. Word recall and visual analogue mood scales were analyzed with linear mixed models including the respective baseline as a covariate and the terms treatment, assessment, respective baseline, treatment × assessment as fixed effects. 

## 3. Results

### 3.1. Participant Demographics

**Figure 2:**
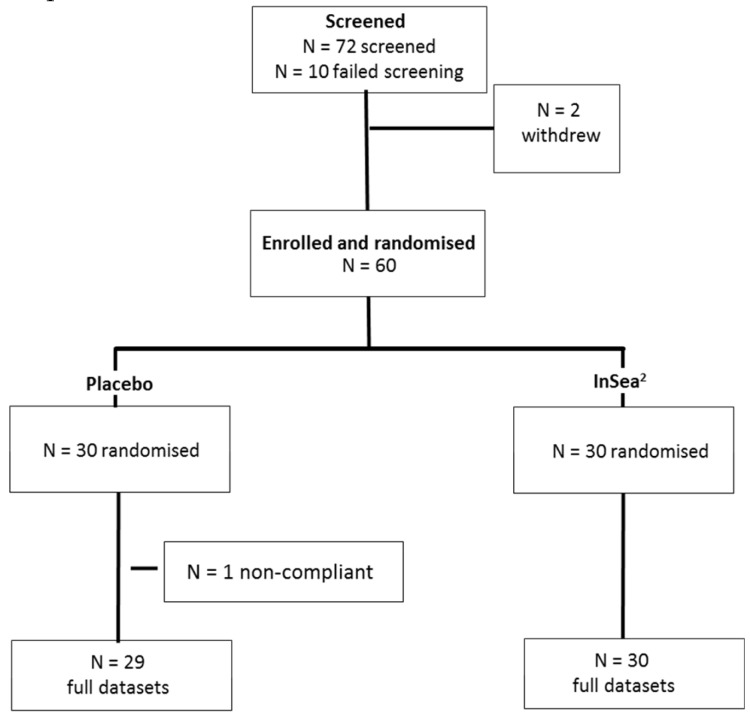
Final subject disposition.

Sixty participants were randomized. Following blinded data screening, one participant was removed from the placebo group as this participant had not engaged with the tasks and produced consistent outliers (see Figure 2). Demographic data for the remaining 59 participants on which the analysis was conducted are presented in Table 2. 

### 3.2. Baseline Performance 

Independent *t*-tests revealed a significant difference at baseline in digit vigilance reaction time *t*(45.725) = 2.055, *p* = 0.046, indicating faster performance on this task in those allocated to receive brown seaweed. As with all other variables, this baseline was entered as a covariate into the analysis of post-treatment scores for that outcome. 

### 3.3. Intervention Effects

#### 3.3.1. Digit Vigilance

**Figure 3:**
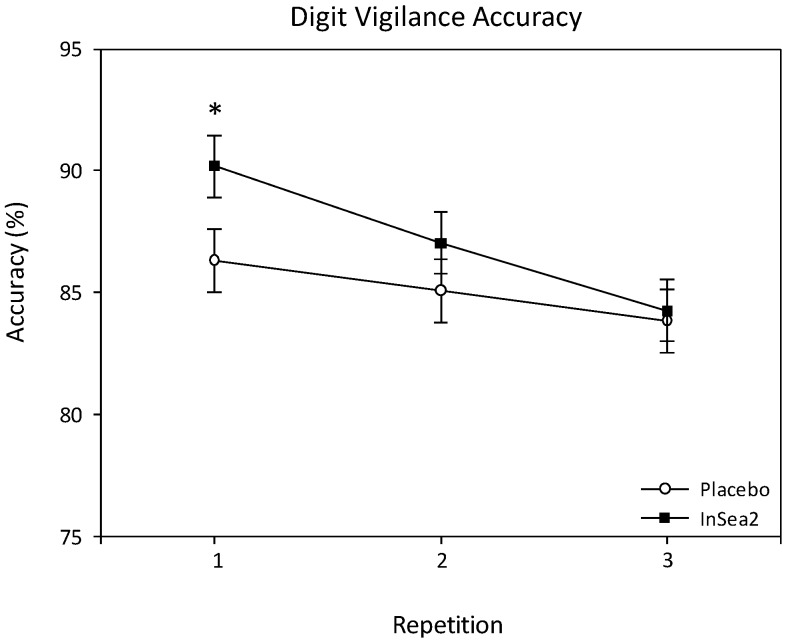
Adjusted means and standard errors (SE) for digit vigilance accuracy collapsed across assessments and * = Treatment × repetition effect, significant difference between placebo and seaweed.

A significant treatment × repetition interaction was observed for digit vigilance accuracy (F(2, 552.240) = 3.377, *p* = 0.035). Pairwise comparisons revealed that those receiving brown seaweed were significantly more accurate than placebo, equating to ~4%, during repetition 1 of the assessments (*p* = 0.039). See Figure 3. There were no other significant effects on digit vigilance accuracy and no significant effects on digit vigilance reaction time.

When examining counts of false alarms, no statistically significant effect of treatment was observed. However, the rate of false alarms was not uniform across participants and treatment groups. In order to explore this effect in more detail, rather than examining counts, a dichotomous variable was created by assigning a value of 1 if the number of false alarms equaled or exceeded a threshold value ranging from 4 to 7, and a value of 0 if the number was lower than the threshold. Threshold analysis was then conducted on high and low false alarm rates using logistic mixed effect models to compare treatment effects for each of the thresholds. In Table 3, therefore, an odds ratio of 1 reflects an equal likelihood of exceeding a threshold when comparing seaweed with placebo and any number above 1 reflects the increased chance of exceeding that threshold following placebo compared to seaweed. This analysis revealed statistically significant differences between placebo and seaweed at rates between 5 and 7, with higher false alarm rates in the placebo group.

#### 3.3.2. Choice Reaction Time

A significant treatment × assessment interaction was observed for accuracy of choice reaction (F(4, 371.063) = 2.489, *p* = 0.043). Pairwise comparisons revealed no significant differences between treatment groups. However, within-group analysis revealed a significant decline in performance at the 120 (*p* = 0.001) and 160 min (*p* = 0.020) assessments in the placebo group when compared to the first assessment post-lunch, which was not apparent in the seaweed group. There were no other significant effects on accuracy of choice reaction and no significant effects on choice reaction time (see Table 4 for means and standard errors for attention tasks).

#### 3.3.3. Simple Reaction Time, Word Recall and VAS Mood Scale Ratings

There were no significant differences between the intervention groups on any outcomes from the simple reaction time task, word recall or VAS mood ratings. 

### 3.4. Treatment Guess

Success of blinding was confirmed via treatment guess at the end of the study. Seventy-six percent of participants in the placebo group and 77% of participants in the seaweed group believed they had received placebo. Chi-squared analysis confirmed this to be a non-significant difference (*p* = 0.942).

### 3.5. Safety Evaluation

All 60 participants were included in the analysis of safety, assessed via reporting of adverse events. No adverse effects of treatment were reported.

## 4. Discussion

The aim of the current randomized, placebo-controlled, double-blind, parallel groups study was to explore the impact of a brown seaweed extract containing phlorotannins (InSea2^®^) on cognitive function when assessed post-prandially following a high-carbohydrate lunch. The results of the current study demonstrate that the seaweed extract was able to improve cognitive performance when compared to placebo. This improvement was observed as significantly greater accuracy on a digit vigilance task when measured during the first repetition of each assessment and attenuation of a decline in choice reaction accuracy seen during later assessments following placebo. Similarly, participants in the placebo group were significantly more likely to produce false alarm rates of 5 and above when compared to the seaweed group. 

This is the first demonstration of cognitive benefits following intake of a seaweed extract and adds to a growing body of literature indicating positive effects of (poly)phenol-rich foods on cognitive performance across various age groups. Acutely, improved continuous performance task accuracy and increased simple finger tapping was observed in middle-aged males following orange juice [23], which is supported by evidence for improvement to digit vigilance accuracy and serial three subtraction performance in elderly adults following curcumin consumption [24]. In healthy young adults, acute intake of purple grape juice was shown to increase a composite score of speed of attention [25], whereas two different extracts of blackcurrant juice were able to either decrease digit vigilance reaction time following a cold-pressed extract or improve accuracy of rapid visual information processing (RVIP) following freeze-drying [22]. Similarly, acute cocoa consumption improved serial three subtraction performance with a dose-specific increase to speed of RVIP [26]. The lack of effects on memory in the current study is unsurprising as previous studies have also failed to find these effects acutely in young [25], middle-aged [23] or elderly participants [24]. However, acute supplementation with wild blueberry was able to improve memory in 7–10 year old children [27], suggesting that there may be specific stages of development which are sensitive to acute manipulation with (poly)phenols. 

A number of mechanisms have been proposed for the action of phenolic compounds on brain function. For instance, cognitive improvements have been attributed to increased cerebral blood flow, which follows on from data showing declines in cerebral blood flow in ageing [28,29] and an inverse relationship between cerebral blood flow velocity and cognitive decline [30]. Importantly, significant benefits to cognition and cerebral blood volume following high flavan-3-ol cocoa have been observed in older adults, with cerebral blood volume positively correlated with cognitive task performance [31]. Increases to cerebral blood flow have also been observed in healthy young adults following (poly)phenols [32,33], demonstrating that these modulations are not restricted to older populations. A related explanation for benefits to cognition is a modulation of glucose metabolism. The seaweed extract employed here has previously been shown to decrease α-amylase and α-glucosidase enzymes in a dose-dependent manner in vitro and to lower blood glucose response in vivo, in rats following starch gavage [17]. Inhibition of these enzymes and the resultant reduced glycemic response to food was therefore predicted to improve cognition, as indicated by previous studies in a range of subjects showing beneficial effects following low glycemic index (GI) foods when compared to high [5,34,35,36]. 

Studies into cognitive effects of GI have, however, produced conflicting results [4,37,38], which are potentially due to differences other than GI between the meals being compared, such as energy density, macronutrient content and micronutrient profile. Data showing modulation of glucose and insulin response following berries [39,40,41] and a reduction in the post-prandial blood glucose response following grape seed extract [42] indicate the potential for phenolic-rich food in improving glucoregulation. However, these foods also contain a high sugar content, which is not the case for seaweed. The addition of a seaweed extract to a meal, therefore, represents a potentially novel approach to lowering glycemic index without significantly altering any other characteristics of the meal. The importance of glucoregulation is demonstrated by studies examining the relationship between impaired glucose tolerance and poor cognition [43]. However, the metabolism of glucose is suggested to be more important than the absolute level and a key mediator in this metabolism is insulin. The importance of insulin to brain function is demonstrated by data showing impairment to cognition in conditions of insulin resistance such as type 2 diabetes. However, whether this is a direct effect or whether improved sensitivity leads to indirect effects via increased supply of glucose to the brain remains to be elucidated. Interestingly, the seaweed extract employed here lowered the insulin incremental area under the curve and increased insulin sensitivity in humans, while the impact on post-prandial glucose levels did not reach statistical significance [19]. Previous studies showing associations between improved cognition and increased insulin sensitivity following short-term supplementation with drugs [44], and cocoa flavan-3-ols [45,46], support the importance of this but this has not been sufficiently explored in relation to acute changes in insulin sensitivity. In order to make a direct link between glycemic control and cognitive effects acutely, it is necessary to measure glycemic and insulinemic response concomitantly with cognitive outcomes and preferably over a large post-prandial period. 

Time course of effects is an important consideration when assessing the impact of GI on cognition given that lowering GI flattens and extends the time course of food related increases in glucose levels. Interestingly, studies that have failed to show positive effects of low GI food have typically assessed effects over a shorter timeframe of up to 105 min [4,47], whereas studies showing cognitive benefits have done so at later post-dose assessments, for instance at 120 to 240 min post-prandially [48,49,50]. In the current study, the cognitive assessments extended from immediately post-meal to 190 min post-meal on the basis of previous data showing reductions in insulin levels across a 180 min post-prandial period following the same seaweed extract [19]. While digit vigilance accuracy was improved during the first repetition of the tasks across all post-meal assessments, examination of the data (Table 2) reveals that this effect was most striking numerically during the final assessment. Similarly, the treatment by assessment interaction on choice reaction accuracy was supported by a within-groups analysis suggesting attenuation of performance deficits following seaweed extract that was restricted to the last two assessments. It is noteworthy that an absence of any effects on word recall may be due to the assessments finishing before the time point at which beneficial effects of low GI on memory function have previously been detected (210 min post-meal) [51].

The current study employed a novel repeated block paradigm designed to be cognitively demanding and to induce fatigue. Furthermore, participants were recruited who self-reported post-meal drowsiness to allow a more sensitive backdrop for effects to be observed following lunch consumption. However, despite decreases in concentration, mental stamina and physical stamina with accompanying increases in mental and physical tiredness following lunch, no effects of intervention on subjective state measures were observed across any of the assessments. In addition, despite anecdotal reports of tiredness and difficulty in completing the tasks, there was no evidence of significant decline in subjective state across the post-lunch assessments. It is possible that the paradigm was simply too intense and participants disengaged from the tasks meaning that no effects on fatigue were apparent. Data showing effects of the seaweed extract on digit vigilance accuracy during the first repetition only may provide support for this suggestion, with effects not being apparent during subsequent repetitions due to a lack of engagement with the task. In order to ensure that the tasks are demanding enough to deplete brain glucose stores it may be preferable for future research to employ a demanding paradigm that has previously been shown to be sensitive to supplementation with cocoa flavan-3-ols [26] and following Panax ginseng, which also led to an acute glucoregulatory effect [52]. Another important aspect of the current study is the use of parallel groups rather than a more robust repeated measures approach. A parallel groups design was chosen in order to reduce the impact of practice and order effects; however, the use of repeated measures would go some way to overcoming issues with individual differences inherent in dietary intervention studies and would greatly strengthen the design.

## 5. Conclusions

In summary, the results presented here are promising and indicate a benefit to cognition following a brown seaweed extract. These effects were shown following a supplement containing the equivalent of 10 g of dried seaweed, suggesting that these benefits could be obtained from dietary intake. Further research is required in order to replicate these findings and to explore any dose-dependent effects of this extract. These studies should investigate the effects on cognition in combination with glucoregulatory measures to verify the importance of this mechanism. In order to provide a full picture of the profile of these effects, future studies should investigate these effects over a post-prandial period that extends beyond 3 h when blood glucose levels have typically returned to baseline in the control condition. Fasting until lunchtime, as in the current study, has previously been shown to trigger increased postprandial hyperglycemia and impaired insulin response after lunch in type 2 diabetes [53], indicating that these findings may have particular relevance to those who skip breakfast. However, it is also important that effects of seaweed on cognition are explored in the absence of this fast since this is not typical behavior for most people. Although not a focus of the current study, recent data also indicates that phlorotannins from brown seaweeds are metabolized in the large intestine with the majority of metabolites not appearing until 8 h post-ingestion [54], which may provide further support for a later testing regime. In addition to glucoregulatory measures, it would be of interest to explore salivary α-amylase. Although inhibition of α-amylase and α-glucosidase by the seaweed extract is known to occur within the digestive tract environment, rather than at the systemic level, the impact of seaweed on salivary α-amylase levels is unknown and this would provide a useful marker for α-amylase and α-glucosidase inhibition if shown. Furthermore, previous studies showing the importance of insulin sensitivity as a predictor of cognitive change following short-term cocoa consumption demonstrate the need to establish the effects of brown seaweed extract over a longer term following repeated intake.

## Figures and Tables

**Table 1:** Treatment composition.

Ingredient	Function	Quantity per Unit	
		Placebo	Seaweed
Brown Seaweed Powder	Active ingredient	0 mg	250 mg
Microcrystalline cellulose	Bulking agent	191 mg	100 mg
Calcium phosphate dibasic	Diluent	287 mg	150 mg
Magnesium stearate	Lubricant	5 mg	5 mg
Caramel	Color	25 mg	3 mg
Capsules composition:			
Titanium dioxide Hypromellose	Opacifier Structure	1.8 mg 94.3 mg	0 mg 0 mg

**Table 2:** Participant demographics.

Measure	Placebo		Seaweed	
	Mean	SD	Mean	SD
*N*	29	-	30	-
Sex (% male)	59	-	50	-
Age (years)	37.9	±16.9	33.1	±14.6
Years in education	17.5	±0.85	17.6	±0.66
Caffeine consumption (mg/day)	165.2	±118.0	170.3	±109.3
Fruit and vegetables (portion/day)	3.78	±1.17	4.37	±1.61
BMI (kg/m^2^)	24.0	±3.05	24.2	±3.55

SD = Standard deviation; BMI = Body mass index.

**Table 3:** Threshold analysis of digit vigilance false alarms.

Threshold	Odds Ratio	Confidence Interval (95%)	Significance (*p*-Value)
≥4	1.61	(0.62, 4.22)	0.33
≥5	2.9	(1.16, 7.27)	0.02
≥6	2.77	(1.0, 7.81)	0.05
≥7	3.41	(1.21, 9.66)	0.02

**Table 4:** Attention scores. Mean and standard errors are presented. Baseline values are raw scores and post-dose values are derived from the linear model with adjustment for the corresponding baseline value.

Measure	Treatment	*N*	Repetition	Baseline	SE	Session Post-Lunch					
						0 min	40 min	80 min	120 min	160 min	SE
Simple Reaction Time (ms)	Placebo	29	1	295.7	±7.11	290.7	294.2	322.2	324.9	342.9	±24.0
			2	-		324.4	370.9	380.5	375.8	394.6	
			3	-		425	406.5	416.8	421.9	391.8	
	InSea2^®^	30	1	276.9	±6.39	293.4	305.7	323.7	322.3	333.4	±23.6
			2	-		316	346.8	342.2	375.8	375.5	
			3	-		359.8	390.9	401.1	443.4	381.6	
Digit Vigilance Accuracy (%)	Placebo	29	1	89.12	±2.61	89.5	88.8	85.4	86.4	81.5	±1.91
			2	-		89.4	86	83.4	85.1	81.7	
			3	-		87.1	84.3	80.5	82.8	84.6	
	InSea2^®^	30	1	93.33	±1.63	91.6	91.2	89.3	90.4	88.4	±1.88
			2	-		90	87.4	88.1	86.9	82.9	
			3	-		87.6	84.6	84.7	82.5	82	
Digit Vigilance Reaction Time (ms)	Placebo	29	1	434.7	±8.89	426.9	439.6	449.8	445.7	448.8	±5.27
			2	-		437	449	446.6	453.6	450.3	
			3	-		438.8	451.6	453.8	453.5	451.6	
	InSea2^®^	30	1	413.4	±5.28	439.3	444.7	449.4	448.7	447.3	±5.18
			2	-		448.6	451.5	456	456.6	458	
			3	-		448.2	464.7	463.6	459.2	459.6	
Digit Vigilance False Alarms (Number)	Placebo	29	1	1.79	±0.43	2.15	2.84	3.74	3.5	3.81	±0.49
			2	-		2.39	3.39	4.01	3.43	4.43	
			3	-		3.7	4.5	4.67	4.19	4.6	
	InSea2^®^	30	1	1.5	±0.36	2.22	2.32	3.05	2.82	3.09	±0.48
			2	-		2.09	2.69	3.25	3.39	3.99	
			3	-		3.52	3.95	3.45	3.59	4.39	
Choice Reaction Time Accuracy (%)	Placebo	29	1	97.6	±0.36	96.7	96.6	95.6	95.9	96.2	±0.55
			2	-		96.6	96.4	96.7	95.2	95.8	
			3	-		96.5	95.8	96.2	95.3	95.4	
	InSea2^®^	30	1	96.2	±0.73	96.3	95.9	96	96	95.9	±0.54
			2	-		95.7	95.9	96.1	95.5	95.9	
			3	-		95.3	95.4	94.4	95.7	96.2	
Choice Reaction Time (ms)	Placebo	29	1	436.4	±14.6	425.1	443.2	466	474.4	475.9	±21.1
			2	-		473.9	476.6	516.3	506.7	524.8	
			3	-		472.4	498.7	479.8	523.2	485	
	InSea2^®^	30	1	406.3	±7.50	429.5	448.5	457.4	468	478	±20.7
			2	-		451.9	478.8	487.5	524.4	489.8	
			3	-		477.9	513.5	498	559.6	496.6	
